# The Transcriptional Regulation of Germinal Center Formation

**DOI:** 10.3389/fimmu.2018.02026

**Published:** 2018-09-05

**Authors:** Shuang Song, Patrick D. Matthias

**Affiliations:** ^1^Friedrich Miescher Institute for Biomedical Research, Basel, Switzerland; ^2^Faculty of Sciences, University of Basel, Basel, Switzerland

**Keywords:** hematopoiesis, transcription factors, B cell development, germinal center (GCs), transcriptional regulation, germinal center development, germinal center maintenance, plasma cell and memory B cell differentiation

## Abstract

Germinal centers (GCs) are essential structures of the humoral immune response, which form in the periphery in response to T cell dependent antigens. During the GC reaction, B cells undergo critical differentiation steps, which ultimately lead to the generation of antibodies with altered effector function and higher affinity for the selected antigen. Remarkably, many of the B cell tumors have their origin in the GCs; thus, understanding how the formation of these structures is regulated or deregulated is of high medical importance. This review gives an overview of the transcription factors that have been linked to the generation of GCs, and of their roles in the process.

## Background to B cell development

B (and T) cells represent a unique model of cellular development, in which cells of multiple differentiation stages can be identified based on surface markers and readily isolated. Owing to these advantages, the lymphoid system has been used widely, beyond immunology, as a developmental paradigm in which the role of transcription factors (TFs) or signaling molecules can be tested experimentally. Several excellent reviews exist that describe in detail how B lymphocytes develop, what regulatory circuits are critical, or the details of GC development (1–11). We will therefore not discuss these aspects in detail, but will only give a high-level overview, and then focus this review on the transcriptional control of GCs formation.

B cells originate and develop in the bone marrow from hematopoietic stem cells (HSCs) that differentiate into progenitor stages of increasingly restricted potential. Once committed to the B lineage, B cell progenitors go through several successive stages, at which key events of their developmental fate take place. In particular, the PreB stage represents the phase during which immunoglobulin (Ig) genes, which code for the antibody molecules, rearrange their DNA segments in order to produce functional genes. The heavy chain rearranges first at the ProB stage, followed by the light chain at the small PreB-II stage. Immature B cells then express IgM at their surface and exit the bone marrow to enter the circulation and move to peripheral lymphoid organs such as the spleen or the lymph nodes. There, marginal zone (MZ) B cells play vital functions in T cell-independent humoral immune responses against blood-borne pathogens, follicular B cells can capture antigen presented by Follicular Dendritic Cells (FDCs) and present it to CD4^+^ follicular T helper cells (T_FH_) that are located around the B cell zone of the developing GC. This is the time during which critical signals, sent by the T_FH_ cells, induce isotype switching (so-called class switching, which exchanges IgM for IgG) and expansion of B cell clones starts. These B cells are called centroblasts and form the dark zone (DZ) of the GC. After several rounds of proliferation, somatic hypermutation begins, a process by which the Ig DNA becomes mutated under the action of activation-induced cytidine deaminase (AID), leading to the generation of diverse clones expressing antibodies with different, potentially higher, affinity for antigen. From there, the B cells (centroblasts) move to the adjacent region called the light zone (LZ), where they express their antibody on the cell surface. GC B cells in the light zone are called centrocytes and are in a near apoptotic state. It is there that selection for the quality (affinity) of the antibody takes place: based on the affinity of the antibody for the antigen, the B cell can be eliminated or rescued and sent back to the dark zone as centroblast for an additional round of mutations, followed by renewed entry into the light zone and further antibody affinity testing. At some point in this dark zone–light zone selection cycle, the B cell expresses a high affinity antibody and can now exit the GC as a plasma cell that secretes high amounts of the antibody, or as a memory B cell that is ready to be reactivated upon future encounter with the antigen.

The rest of this review will put the emphasis on the transcriptional control of the formation and function of GCs, and highlight in particular TFs that are essential.

## Transcription factors regulating GC formation

### GC initiation

**Figure 1:**
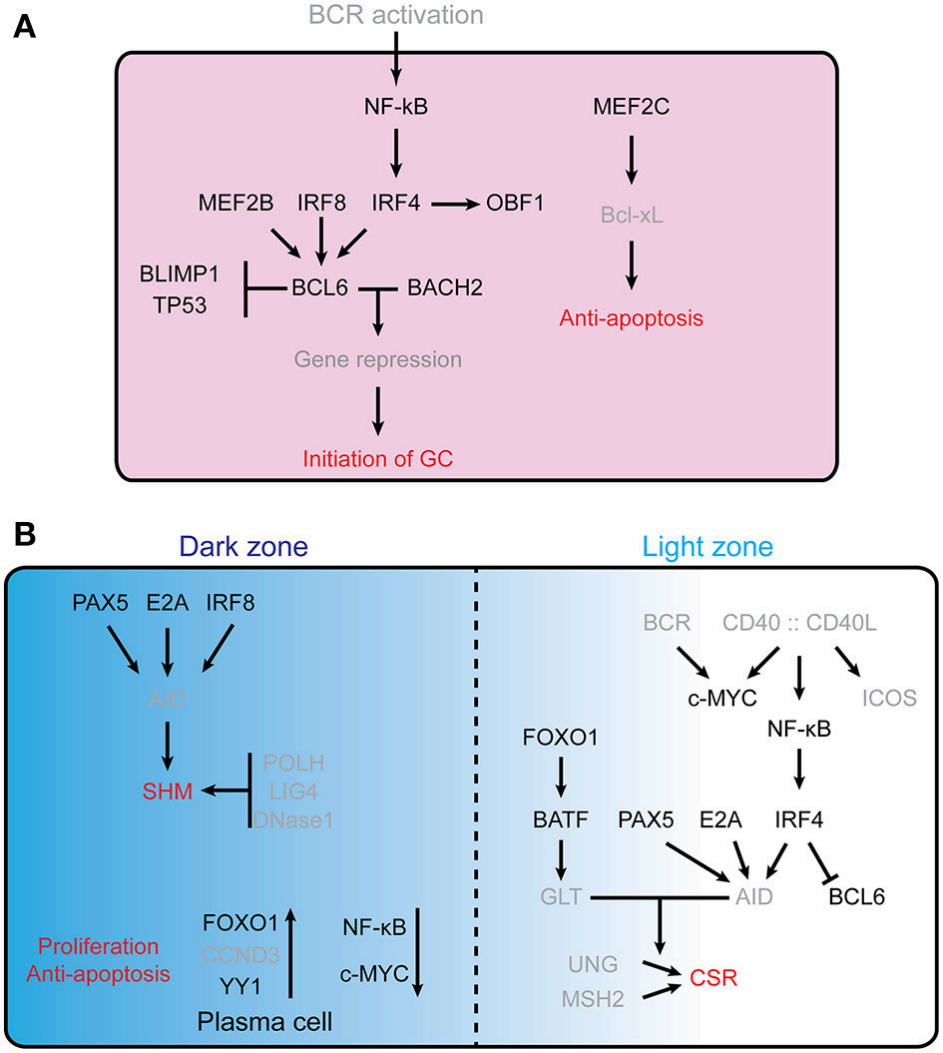
Transcription factors controling GC formation. **(A)** Initiation of the GC reaction in follicular B cells. For clarity, TFs are indicated in black, while other molecules (e.g., receptors, cytokines, etc…) are in gray. B cell lymphoma 6 (BCL6) is essential for the initiation of germinal center, MEF2B, IRF8, IRF4, BLIMP1, and TP53 are involved in regulating the expression of *Bcl6*. BCL6 and Bach2 cooperatively (12) repress gene expression and thus allow the establishment of the germinal center B cell program. MEF2C is required for B cell survival post-antigen stimulation by upregulating the Bcl2l1. **(B)** Schematic representation of the dark zone and light zone of the GC. The different TFs involved are indicated, as well as some of the processes regulated (SHM, Proliferation, anti-apoptosis, CSR), see text for further details. In the GC DZ, AID is a key enzyme for SHM; its expression is controlled by PAX5, E2A, and IRF8. POLH, LIG4, and DNaseI are required for SHM and are highly expressed in DZ B cells. FOXO1 is a key factor for maintaining the GC DZ B cell program, CCND3 is preferentially expressed in GC DZ B cells and YY1 is required for GC DZ B cell proliferation and survival. NF-κB signaling and c-Myc are not essential for GC DZ B cells. In the GC LZ, CD40 signaling stimulated NF-κB further stimulates IRF4 expression, which suppresses *Bcl6* gene (13, 14). PAX5, E2A, and IRF4 are key factors in regulating AID level. BATF, a downstream target of FOXO1, regulates germline transcripts (GLTs) in centrocytes. GLT levels are highly correlated with accessibility of AID in CSR.

Initiation of the GC reaction involves activation of the B cell receptor (BCR) by antigen engagement, followed by interaction of these B cells with antigen presenting cells and T_FH_ cells, which provide further activation signals (2, 3). Figure 1 summarizes the molecular networks regulating initiation and function of the germinal centers reaction.

Transcription factors that are downstream of the BCR, such as the transcription coactivator OBF1 (a.k.a. OCA-B, or Bob1), a B cell-specific coactivator for the octamer transcription factors OCT1 and OCT2, are critical for GC formation (15–18). Mice deficient in *Pou2f2* (encoding OCT2), *Pou2af1* (encoding OBF1) or both showed complete lack of GCs (19). The underlying molecular mechanism is not clear yet, and the target genes of OBF1/OCT2 in the context of the germinal center reaction are largely unknown, although Spi-B which itself is required for GCs (20, 21) has been identified as a downstream target of OBF1 (22). Moreover, in CD4^+^ T cells OBF1 and OCT1/OCT2 directly bind to the promoter region of *Bcl6* and activate its transcription, thereby promoting the development of T_FH_ cells (23). The putative role of these factors in regulating *Bcl6* expression in early GC B cells remains to be investigated.

BCL6 is a zinc finger TF that is essential for germinal center formation, as *Bcl6*-null mice completely lack GCs and affinity maturation (3, 24). During the early phase of the GC response, antigen stimulated B cells rely on T_FH_ cells for differentiation into GC B cells, and interaction between T_FH_ and B cells leads to the upregulation of BCL6 (25). Moreover, the upregulation of BCL6 leads to stabilized conjugation between B and T_FH_ cells, creating a positive feedback loop that enhances the GC formation program (3, 25). Failure in BCL6 upregulation prevents B cells from entering GC clusters and impairs the upregulation of CXCR4, a chemokine receptor expressed on germinal center DZ B cells that is critical for the maintenance of GC structural integrity (25).

IRF4 is required at the early stage of GC formation. In transplantation experiments, *Irf4*^−/−^ B cells fail to differentiate into GC B cells (26). Conditional knockout of *Irf4* by CD19cre which deletes from early B cells onwards leads to impaired GC formation (26). In contrast, once GCs have formed or initiated, IRF4 is no longer needed, as conditional knockout by Cγ1cre which deletes in already formed GC cells has minimal effects on GC differentiation (27). These results suggest that IRF4 is required for the very early phase upon T-cell-dependent antigen stimulation. Additional evidence supporting this idea is the rapid upregulation of IRF4 following BCR stimulation (28). Moreover, IRF4 is involved in modulating the expression of BCL6 and OBF1, which both are key factors for GC initiation (3, 26). Taken together, IRF4 plays an important role in the early initiation phase of GC formation, possibly by regulating the induction of *Bcl6* and *Pou2af1*.

IRF8 was reported to upregulate BCL6 and AID levels in GC B cells (29, 30), and it was shown to promote GC B cells survival by regulating the expression level of MDM2 (31). However, deletion of IRF8 in B cells did not affect GC formation (32). Moreover, IRF8 is involved in the regulation of the BCL6-related transcriptional program in GC cells by directly interacting with BCOR (B cell lymphoma 6 corepressor) and BCL6. In transactivation assays, IRF8 augments the transcription repressive activity of BCL6 (33).

MEF2C is required for the proliferation and survival of B cells upon antigen receptor stimulation by upregulating the expression level of *Bcl2l1* (encoding the Bcl-xL protein) and several cell cycle related genes (34). Specific deletion of *Mef2c* in B cells leads to reduced proliferation and increased cell apoptosis upon anti-IgM stimulation. However, the responses are normal in the case of LPS, CD40, IL4, BAFF and RP105 stimulations. By histological examination, reduced number of GC follicules are observed in the spleens of *Mef2C*^*fl*/*fl*^-CD19cre mice immunized with sheep red blood cells (SRBC) (34). MEF2B, another member of the MEF2 family, has been found to be mutated in ca. 11% of diffuse large B cell lymphoma (DLBCL), which are GC-derived tumors (35). MEF2B directly activates *Bcl6* transcription by binding to the regulatory region 1 kb upstream of the *Bcl6* gene transcription start site (35). Mutation of the MEF2B binding motif in the *Bcl6* gene promoter abrogates *Bcl6* transcription activity in cotransfection assays in 293T cells. Furthermore, knockdown of MEF2B protein by shRNAs leads to downregulation of BCL6 and upregulation of BCL6 target genes. These data suggest that MEF2B plays an important role in early GC formation by modulating *Bcl6* expression (35, 36).

BATF is a transcription factor of the AP-1 family, which is involved in GC structure establishment and class switch recombination. *Batf*^−/−^ mice failed to develop normal GC structures when immunized with SRBC, as characterized by a lack of CD95 or GL7 positive B cells (37). *Batf*-null T_FH_ cells lack expression of the chemokine receptor CXCR5, which is essential for GC structure integrity. Additionally, the expression of *Bcl6* and *c-Maf*, both of which are important factors for T_FH_ cells development, is downregulated in absence of BATF (37).

c-MYC is another TF indispensable during the early phase of germinal center formation. Its expression is induced already 1–2 days after immunization (38) and it is required for GC maintenance, as conditional deletion of *c-Myc* by Cγ1cre leads to impaired GCs (39).

### GC development

The dark zone and the light zone of the GC are organized by the expression of the chemokine receptors CXCR4 and CXCR5, respectively (40). Thus, one can expect that TFs critical for CXCR4 and CXCR5 expression will be important for GCs.

#### GC dark zone

The germinal center DZ is characterized by an interconnected network of CXCL12 expressing reticular cells and compactly filled with rapidly proliferating centroblasts (41).

FOXO1 is highly expressed in human and mouse GC B cells, and its expression is largely specific to DZ B cells (with also some expression in naïve B cells) (42). Like in *Cxcr4*^−/−^ mice, GCs from *Foxo1*^*fl*/*fl*^-Cγ1cre mice completely lack a DZ structure, while the differentiation of plasma cells is normal (42, 43). *Foxo1*-null GCs lack proper structural polarization and show an even distribution of the FDC network (42). FOXO1, together with BCL6, represses the expression of B lymphocyte induced maturation protein 1 (BLIMP1), a key factor promoting differentiation of GC B cells into plasma cells, which is encoded by the *Prdm1* gene. By binding to the *Prdm1* promoter region, FOXO1 and BCL6 maintain the germinal center DZ program (42).

*Bcl6*-null GC precursor B cells fail to upregulate the expression of CXCR4 (25), which is a crucial chemokine receptor for GC DZ B cells. c-MYC is required throughout the early and late initiation phases of GC formation, but is not expressed in the proliferating DZ B cells (3), where it is repressed by BCL6 (38).

YY1 is required for GC B cell proliferation and GC development at least partly by modulating cell apoptosis (44). Deletion of *Yy1* specifically in GC B cells leads to a significant decrease in the number of DZ B cells, and elevated cell apoptosis (44).

##### Somatic hypermutation (SHM)

SHM generates a wide repertoire of affinities toward specific antigens, and mainly takes place in the DZ (45), although some extrafollicular SHM has been reported in transgenic mice deficient in the ability to establish GCs (46). AID, encoded by the *Aicda* gene, is the enzyme responsible for SHM and class switch recombination (47, 48). AID deaminates cytidines in DNA (49–54), followed by error-prone repair involving different DNA repair factors and ultimately leading to the introduction of somatic mutations (55). Thus, transcription factors which affect the expression of *Aicda* and DNA-damage tolerance related genes should be important for SHM. E proteins (56), PAX5 (57) and IRF8 (29) have been associated with positive regulation of *Aicda* transcription.

FOXO1 is involved in SHM by affecting the protein level of AID: *Foxo1*-null GC B cells show reduced level of AID enzyme, while mRNA level of *Aicda* is unchanged. Therefore, *Foxo1*-null GC B cells carry lower level of mutations in Ig locus than control cells (58).

*Irf8* mRNA level peaks in centroblasts, and IRF8 regulates SHM by modulating the expression of *Aicda* and *Bcl6*: knockdown of IRF8 by siRNA leads to decreased transcription of *Aicda* and *Bcl6* (29). By ChIP, IRF8 binds to the promoter regions of *Aicda* and *Bcl6* in both human and mouse B cells. Furthermore, luciferase assays showed that IRF8 directly regulates the transcription of *Aicda* and *Bcl6* in HeLa cells cotransfected with an IRF8 expression vector and a reporter containing promoter regions of *Aicda* or *Bcl6* (29). Moreover, IRF8 promotes GC B cells survival by regulating the expression level of MDM2 in the case of DNA damage (31).

*Aicda* expression is significantly reduced in activated B cells in which the helix-loop-helix factor ID3 is ectopically expressed. The possible mechanism is that ID3 inhibits the DNA-binding activity of E-proteins which activate the expression of *Aicda* (56, 59).

#### Light zone

Three crucial B-cell developmental processes take place in the GC light zone: (i) selection of B cells that produce high-affinity antibodies, (ii) CSR, and (iii) initiation of centrocytes differentiation into plasma cells or memory B cells (9).

After rapid expansion in the DZ, B cells migrate to the LZ where those carrying high affinity B cell receptor genes are selected. The BCR pathway plays a fundamental role in this process: BCR signaling leads to phosphorylated AKT, and activated AKT further phosphorylates FOXO1 which then relocates from the nucleus to the cytoplasm (60). CD40 stimulation leads to NF-κB-mediated upregulation of IRF4 (13), which in turn represses *Bcl6* transcription (61). Together, these coordinated actions terminate the dark zone-associated transcriptional programme and allow establishment of the LZ transcriptome (13).

c-MYC is absent in most GC B cells, however, its expression is induced in high affinity BCR presenting GC B cells when receiving help from T_FH_ cells, in a process that requires both BCR and CD40 signaling (60). In addition to the requirement of c-MYC activity during the initial stage of GC formation, c-MYC is needed for GC maintenance in the late GC response (38). With the help from T_FH_ cells, c-MYC is transiently induced and upregulated in a small fraction of high affinity BCR expressing GC B cells within the LZ compartment. The Omomyc protein inhibits c-MYC function by antagonizing its DNA binding activity (62). Specific inhibition of c-MYC function by Doxycyclin-induced Omomyc expression in late GC B cells (10 days post-immunization by SRBC) leads to reduced GC size, indicating that c-MYC is required for GC maintenance once GCs are established (38).

##### Affinity maturation

FOXO1 is necessary for effective antibody affinity maturation: SHM frequency is comparable between WT and *Foxo1*-null GC B cells, but a severely decreased number of GC B cells harboring high affinity antibodies is observed in *Foxo1*-null GCs (42). Furthermore, *Foxo1*-null GC B cells have a lower level of cell surface BCR and are ineffective in activating T_FH_ cells in the LZ; this leads to lower stimulation of T_FH_ cells in the GC microenvironment and reduced production of IL-21, a cytokine that is vital for antibody affinity maturation (58). Thus, FOXO1 regulates antibody affinity maturation through both antigen presentation and T_FH_ cell activation (42, 58).

c-MYC is transiently induced in LZ B cells after receiving help from T_FH_ cells (38); selected GC B cells with induced c-MYC present high affinity BCR on the cell surface, and subsequently migrate into the DZ for the next iteration of proliferation and SHM (38, 39). However, in *Foxo1*-null GC B cells, the expression level of c-MYC is downregulated even under the help from T_FH_ cells. BATF, which controls the expression of *Aicda*, is another TF downregulated in the absence of *Foxo1* (58).

#### Class switch recombination

Like SHM, CSR also requires the expression of *Aicda* (47, 48). However, CSR depends on a different domain of the AID protein (63, 64). It is worthwhile mentioning that CSR already takes place before the GCs are formed, following B cell activation (65, 66). Much of the knowledge about CSR and the required factors originates from *in vitro* B cell activation experiments.

IRF4 was shown to regulate CSR in CD40 and IL4 stimulated B cells (27, 67). In the absence of IRF4, the *Aicda* expression level is decreased (67), and CSR is impaired (67). However, the expression of other genes important for CSR, such as Ung or Msh2 remains normal in *Irf4*^−/−^ B cells. Therefore, the CSR defects in *Irf4*-null B cells seem to mainly reflect the impaired *Aicda* expression (67).

FOXO1 deficiency results in impaired class-switching: the compartment of *Igg1*-switched B cells in *Foxo1*-null GCs is heavily reduced, with accumulation of IgM^+^ GC B cells. Yet, the expression level of *Aicda* is similar between WT and *Foxo1*^*fl*/*fl*^-Cγ1cre GC B cells (42). In addition, *Foxo1*-null GC B cells display significantly lower expression of germline transcripts (GLTs) across the Ig locus. GLTs coincide with open chromatin and allow the exposure of switch regions to AID, which in turn induces single-strand DNA breaks through which class switch recombination is accomplished (68, 69). Thus, lower levels of GLTs correlate with reduced accessibility of AID toward the class switch regions. At the molecular level, FOXO1 possibly modulates GLT and post-switch transcripts by binding to I-mu, the 3′ IgH enhancer and a super-enhancer (70) in the Ig locus. Moreover, the transcription factor BATF, which is necessary for the expression of Ig GLTs and subsequent CSR (37), is downregulated upon FOXO1 depletion (42).

PAX5 binds to the promoter region of *Aicda* and activates its expression. Overexpression of PAX5 in a ProB cell line induces the expression *Aicda*, while ID2 has an antagonizing effect on this induction. Moreover, PAX5, E2A, and AID directly interact with each other and form a complex, which contributes to directing AID to the *Igh* locus for CSR (71). In addition, ID2 and ID3 negatively regulate CSR by repressing *Aicda* expression (56, 57).

BATF directly controls the expression of *Aicda*. By ChIP-seq and EMSA, BATF was shown to bind to the regulatory region of *Aicda*. In line with this, the expression level of *Aicda* is downregulated in *Batf*^−/−^ mice (37). Consequently, production of isotype switched antibodies is almost completely missing, although IgM production upon T-cell-dependent or -independent antigen stimulation is still normal in *Batf*^−/−^ mice (37). Moreover, *Batf*^−/−^ mice display a reduction in GLTs from different isotypes, except those from μ-chain. Germline transcription initiated by switch region (I) region promoters, located upstream of the different constant heavy chain exons, is required for AID targeting and successful CSR (8, 54). Taken together, BATF regulates CSR by modulating the expression of *Aicda* and GLTs from the Ig locus.

#### TFs controlling the migration of cells between dark and light zone

The LZ-to-DZ transition is mainly driven by high affinity antibody presentation on the surface of GC B cells and the subsequent help from T_FH_ cells toward high affinity antibody LZ cells (58, 72–74).

*Foxo1*-null GC B cells showed reduced level of surface BCR and Igβ when compared to WT cells (58). Therefore, reduced antigen presentation on *Foxo1*^−/−^ GC LZ B cells fails to effectively activate T_FH_ cells, resulting in lower number of T_FH_ cells and decreased production of IL-21, an important cytokine for GC B cell differentiation and affinity maturation promoted by T_FH_ cells (75, 76). Moreover, *Foxo1*-null GC LZ B cells express less IL21R, further abrogating the ability to receive help from T_FH_ cells (BCR and CD40 signal). Therefore, the LZ-to-DZ migration of selected cells is impaired in the absence of *Foxo1* (58) and *Foxo1*-null GC B cells are trapped in the LZ compartment. Furthermore, *Foxo1*-null GC B cells showed lower proliferation rate in the LZ compartment, in spite of harboring a high affinity BCR, indicating defects in cyclic reentry mediated by help from T_FH_ cells which is coupled with the LZ-to-DZ migration (58).

Interaction between high affinity BCR expressing LZ B cells and T_FH_ cells leads to activation of c-MYC expression (60), which promotes cyclic reentry and LZ-to-DZ migration (58).

### Differentiation: memory/plasma cell fate decision

**Figure 2:**
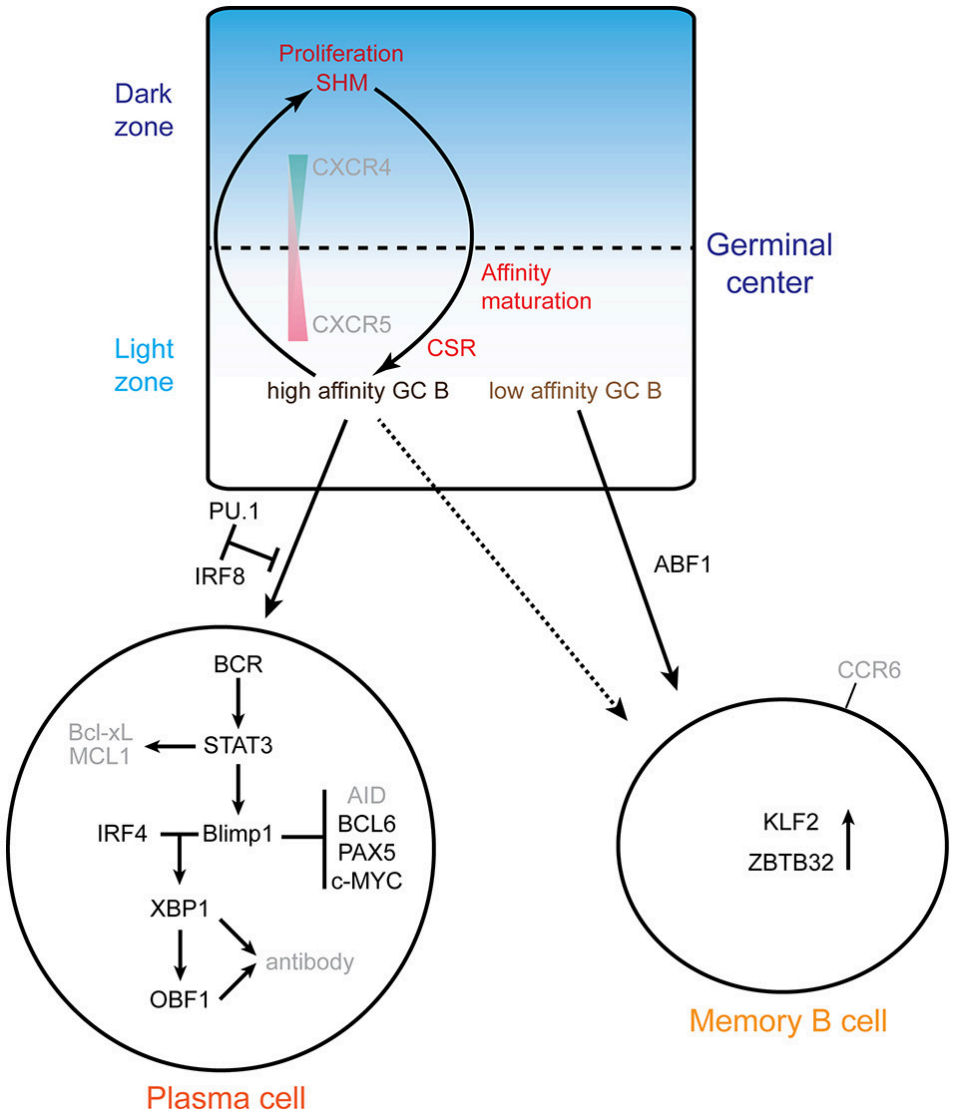
Exit from the GC: memory vs. plasma cells. The top scheme highlights the cycling of B cells between the dark zone and the light zone. The gradients of the chemokine receptors CXCR4 and 5 are indicated, as well as the processes involved. GC B cells proliferate and undergo somatic hypermutation (SHM) in the GC DZ, and then migrate to the light zone where the affinity of mutated BCRs is selected. B cells with high affinity BCR are selected to reenter the DZ for several rounds of SHM or can differentiate into plasma cells. Memory B cells are mainly originated from B cells with low affinity BCR. The alternative fates of B cells exiting the GC are depicted at the bottom: plasma cells or memory B cells. The main networks that have been identified are indicated. In the plasma cell differentiation pathway, PU.1 and IRF8 negatively regulate plasma cell differentiation. IRF4 and BLIMP1 form the central axis in establishing plasma cells. BLIMP1 further suppresses the expression of *Aicda, Bcl6, Pax5* and *c-Myc* genes and finally terminates the GC program. BLIMP1 positively regulates *Xbp1* expression, makes the cells ready for antibody production and secretion. ABF1 promotes memory B cell differentiation and inhibits plasma cell differentiation. ZBTB32 and KLF2 are highly expressed in memory B cells.

Figure 2 summarizes the molecular networks involved in plasma cell and memory B cell differentiation.

Transient and low expression of *Irf4* leads to the expression of *Bcl6* and *Pou2af1* during the early phase of GC formation, while sustained and high level expression of *Irf4* is required for plasma cell differentiation (26). *Irf4* expression is induced to a high level in the fraction of LZ B cells which present high affinity antibodies (26, 28). BLIMP1 is a transcriptional repressor that is essential for plasma cell development: *Prdm1*-deficient mice cannot produce plasma cells and overexpression of BLIMP1 is sufficient to induce plasmablast differentiation (77, 78). *Prdm1* is a downstream target of IRF4, and BLIMP1 can further increase the level of IRF4, thus reinforcing the plasma cell differentiation program in a feed forward loop (26, 79). Morever, BLIMP1 represses the expression of *Aicda* (79) and *Abf-1* (80).

BLIMP1, together with IRF4, acts upstream of X-box binding protein 1 (XBP1), a transcription factor that is essential for upregulation of the secretory apparatus required for antibody production in plasma cells (27, 81, 82). PAX5 represses XBP1 and thus prevents plasma cell differentiation (83). Conversely, downregulation of *Pax5* by BLIMP1 is a necessary step for induction of *Xbp-1* and activation of the plasma cell program (81, 84). However, in the absence of IRF4, BLIMP1 alone is not sufficient for plasma cell differentiation. Moreover, BLIMP1 is required for post-transcriptional regulation of XBP1 mRNA, by which the active form XBP1 protein is generated (85). XBP1 directly regulates *Pou2af1* expression in plasma cells (86), which might be important for IgG production, since OBF1 and OCT2 are required for normal IgG expression (87). *Irf4*-deficient (Cγ1cre) mice completely lack CD138^+^ (aka Syndecan1^hi^) plasma cells in spleen, peripheral blood and bone marrow (27). In addition to the important yet mechanistically unclear role of OBF1 in the early development of GC formation, this factor is required for antibody production (both unswitched and switched isotypes) in T-dependent antigen stimulation and normal antibody secreting cell differentiation, as the number of antibody-secreting Syndecan1^hi^ cells is dramatically reduced in absence of OBF1 (88). Moreover, OBF1 is required for the induction of *Prdm1* (88). Taken together, IRF4 and BLIMP1 function together to drive the transition from GC B cells to plasma cells by repressing the GC program and enhancing the plasma cell differentiation program.

In contrast, IRF8 together with PU.1 inhibit the GC B cell differentiation toward plasma cells, suggesting that the balance between IRF4 and IRF8 may be critical for the fate of B cells at this developmental transition (89). In addition, STAT3 regulates the differentiation of plasma cells possibly by promoting cell survival through activating the expression of pro-survival genes such as *Bcl2l1* and *Mcl1* (90, 91).

It is not resolved yet which transcription factor(s) play a major role in regulating the differentiation of memory B cells (92); however, recent evidence suggests that memory B cells originate from the low affinity compartment of the LZ (92, 93). Comparison of the transcriptome profiles from high- and low-affinity BCR expressing GC B cells in the LZ compartment showed that genes involved in DZ maintenance and cyclic reentry are downregulated in low-affinity fractions (with switched isotype), where *Bach2* and *Pax5* are upregulated (93). As indicated above, upregulation of these transcription factors blocks plasma cell differentiation (94–96).

BACH2 regulates LZ B cells to commit to the memory B cell differentiation path in a dosage dependent manner, as complete knockout or haploinsufficiency of *Bach2* lead to lower memory B cell differentiation (93). Furthermore, T_FH_ cell interaction and affinity maturation in LZ compartment are negatively correlated with *Bach2* expression, thus confirming that memory B cells are generated from low affinity fraction in the LZ compartment (92, 93).

Activated B cell Factor 1 (ABF-1), a helix-loop-helix TF predominantly expressed in memory B cells, blocks the plasma cell differentiation program (80). An inducible ABF-1-ER mouse model demonstrated that induction of ABF-1 promotes GC formation and memory B cell differentiation (80, 97). ZBTB32 and KLF2 are two factors expressed in memory B cells and which inhibit the GC response (98). They have been associated with memory B cells (99), but further mechanistic studies are needed to understand their specific role. BCL6 is a known inhibitor of plasma cell differentiation which directly represses the expression of *Prdm1* (100, 101). STAT5 directly modulates the expression level of *Bcl6*, which in turn directly represses *Prdm1* expression, and thus promotes memory B cell differentiation.

## Outlook and open questions

A central theme is that often multiple distinct TFs act in concert to promote, or repress, specific steps of GC development. Some factors, such as BCL6 or BLIMP1, are considered to be master regulators for GC or plasma cell development, respectively. Yet, many other factors have been shown to be important (FOXO1, IRF4) or sometimes essential (OBF1, OCT2). In most cases, however, additional mechanistic studies are required to precisely understand how these factors fit in the overall regulatory circuitry. Moreover, it is often not clear how the TFs perform their function in this biological paradigm: which co-activators or co-repressors are involved and what epigenetic regulators are required?

Finally, intensive investigations have been conducted to understand the interaction between high affinity BCR expressing GC B cells and T_FH_ cells, yet little is known about how GC B cells are determined by transcriptional regulators to proceed with the LZ-to-DZ migration, or commit to the plasma cell differentiation cascade.

## Author contributions

SS wrote the manuscript and PM corrected the manuscript.

### Conflict of interest statement

The authors declare that the research was conducted in the absence of any commercial or financial relationships that could be construed as a potential conflict of interest.
